# Liquid–liquid phase separation of the prion protein is regulated by the octarepeat domain independently of histidines and copper

**DOI:** 10.1016/j.jbc.2024.107310

**Published:** 2024-04-22

**Authors:** Janine Kamps, Verian Bader, Konstanze F. Winklhofer, Jörg Tatzelt

**Affiliations:** 1 Department Biochemistry of Neurodegenerative Diseases, Institute of Biochemistry and Pathobiochemistry, Ruhr University Bochum, Bochum, Germany; 2 Cluster of Excellence RESOLV, Bochum, Germany; 3 Department Molecular Cell Biology, Institute of Biochemistry and Pathobiochemistry, Ruhr University Bochum, Bochum, Germany

**Keywords:** prion disease, copper, neurodegenerative disease, tryptophan, histidine, liquid-liquid phase separation, octarepeat, cation-pi interactions

## Abstract

Liquid-liquid phase separation (LLPS) of the mammalian prion protein is mainly driven by its intrinsically disordered N-terminal domain (N-PrP). However, the specific intermolecular interactions that promote LLPS remain largely unknown. Here, we used extensive mutagenesis and comparative analyses of evolutionarily distant PrP species to gain insight into the relationship between protein sequence and phase behavior. LLPS of mouse PrP is dependent on two polybasic motifs in N-PrP that are conserved in all tetrapods. A unique feature of mammalian N-PrP is the octarepeat domain with four histidines that mediate binding to copper ions. We now show that the octarepeat is critical for promoting LLPS and preventing the formation of PrP aggregates. Amphibian N-PrP, which contains the polybasic motifs but lacks a repeat domain and histidines, does not undergo LLPS and forms nondynamic protein assemblies indicative of aggregates. Insertion of the mouse octarepeat domain restored LLPS of amphibian N-PrP, supporting its essential role in regulating the phase transition of PrP. This activity of the octarepeat domain was neither dependent on the four highly conserved histidines nor on copper binding. Instead, the regularly spaced tryptophan residues were critical for regulating LLPS, presumably *via* cation–π interactions with the polybasic motifs. Our study reveals a novel role for the tryptophan residues in the octarepeat in controlling phase transition of PrP and indicates that the ability of mammalian PrP to undergo LLPS has evolved with the octarepeat in the intrinsically disordered domain but independently of the histidines.

A conformational transition of the cellular prion protein (PrP^C^) into a β-sheet-rich isoform, denoted scrapie prion protein, causes prion diseases in humans and some other mammals. PrP^C^ is composed of a large intrinsically disordered N-terminal and a structured C-terminal domain, containing three alpha-helical regions and a short, two-stranded beta-sheet (1, 2, 3). Considering the evolution of PrP^C^, it is interesting to note that the three-dimensional structure of the globular structured C-terminal domain of human (amino acid (aa) 121–230), chicken (aa 121–225), turtle (aa 121–225), and *Xenopus* *laevis* (aa 90–222) PrP^C^ shows extensive similarities, suggesting a conserved activity (4). In contrast, the N-terminal unstructured domain (N-PrP) is highly variable among tetrapod classes. This is in line with the observation that intrinsically disordered domains often evolve more rapidly than well-structured protein domains (5). Specifically, mammalian N-PrP contains glycine-rich octarepeats with conserved tryptophan, proline, and histidine residues in this region (6), while amphibian-PrP is devoid of any repeats and histidine residues (7) (Fig. 1, *A* and *B*). The mammalian octarepeat region is of particular interest in prion biology. Most importantly, extra octarepeat insertions in the PrP gene are associated with inherited prion diseases in humans (8, 9, 10, 11, 12) and a neurological illness in transgenic mice (13). On the molecular level, the four histidines of the octarepeat domain mediate an interaction of PrP with divalent metal ions such as copper, zinc, nickel, and manganese ions (14, 15, 16, 17, 18). The effects of copper binding and the histidines on conformation and function of PrP^C^, as well as on the formation of scrapie prion protein, have been studied in more detail (19, 20, 21, 22, 23, 24, 25, 26, 27).

**Figure 1:**
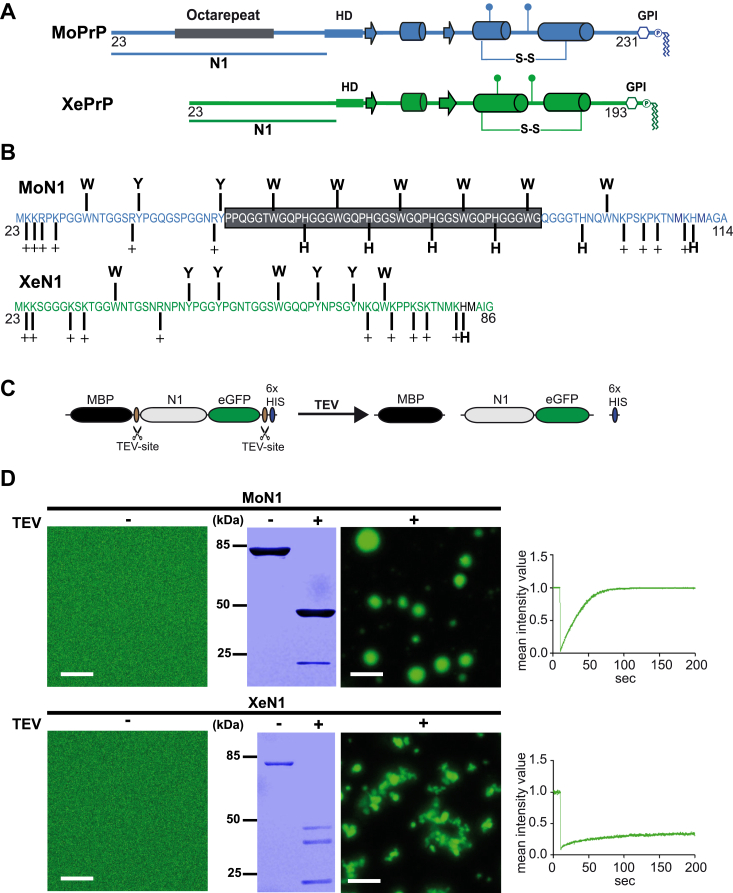
**MoN1 undergoes LLPS, while XeN1 forms undynamic assemblies.***A*, schematic presentation of MoPrP (*blue*) and XePrP (*green*). Mouse octarepeat: *gray box*; *arrows*: beta strands; *tubes*: alpha helices. *B*, amino acid sequence of MoN1 and XeN1. Aromatic amino acids, positively charged amino acids, and histidines are highlighted. The presence of the histidine in XeN1 is due to the 3F4 epitope that was inserted to allow antibody detection of XePrP. Neither MoN1 nor XeN1 contains amino acids with negatively charged side chains. *C*, schematic presentation of the experimental approach. After expression in *Escherichia coli* and purification, phase separation is induced by removing the N-terminal maltose-binding protein (MBP) and C-terminal His tag (6x HIS) by incubation with TEV protease. *D*, MoN1-GFP and XeN1-GFP (10 μM in 10 mM Tris, pH 7.4) were analyzed by laser scanning microscopy before (TEV-) and after addition of TEV protease (TEV+). TEV protease cleavage was performed for 1 h. The scale bar represents 10 μm (*left panels*). An aliquot of each sample (4.5 μg) was analyzed in parallel by SDS-PAGE and Coomassie brilliant blue staining (*middle panels*). Protein mobility within the droplets was measured by fluorescence recovery after photobleaching (FRAP). After 10 s of baseline recording (pre-bleach), a small area of interest (AOI) was photobleached. The average normalized fluorescence intensity of three AOIs was plotted over time (*right panels*). C-C: disulphide bond; GPI: glycosylphosphatidylinositol anchor; HD, hydrophobic domain; LLPS, liquid-liquid phase separation; TEV, tobacco etch virus.

Several proteins linked to neurodegenerative diseases have the ability to form biomolecular condensates *via* LLPS, leading to a concept that condensates are precursors of neurotoxic protein aggregates (28, 29, 30, 31, 32, 33, 34, 35, 36). The mammalian PrP and N-terminal fragments thereof have been reported to undergo LLPS, pointing to a critical role of the N-terminal unstructured domain in regulating the phase separation of PrP (37, 38, 39, 40, 41, 42, 43). Indeed, in many cases, the activity to undergo LLPS is associated with the presence of intrinsically disordered domains with low complexity sequences that do not form stable folded structures (44, 45). Formation of biomolecular condensates of these domains is often driven by electrostatic (46, 47), π–π, cation–π (48, 49, 50, 51, 52), and hydrophobic (53) interactions. We have previously shown that two polybasic motifs located in the N- and C-terminal regions of N-PrP are required for LLPS, and since N-PrP contains no negatively charged residues, this effect is presumably due to cation–π interactions (42). Interestingly, the two polybasic motifs are highly conserved in all tetrapods including amphibian PrP (7, 54). To specifically investigate the role of the mammalian octarepeat in regulating phase transitions, we performed a comparative analysis of the unstructured N-terminal domains of mammalian PrP (MoN1) with that of amphibian PrP (XeN1), which lacks a repeat domain. Our study revealed that the mammalian octarepeat is essential to promote the formation of biomolecular condensates *via* LLPS and to prevent protein aggregation. Strikingly, however, this activity did not depend on the histidines or on copper binding but rather on the regularly spaced tryptophan residues.

## Results and discussion

### Mouse N1-PrP but not *Xenopus* N1-PrP undergoes LLPS

To specifically address the role of the mammalian octarepeat in regulating phase transition of the intrinsically disordered N-terminal domain of PrP, we compared LLPS of *Mus musculus* N1 (MoN1) with that of *Xenopus laevis* N1 (XeN1), a naturally occurring N1-PrP lacking a repeat region (Fig. 1, *A* and *B*). Importantly, XeN1 harbors the two polybasic motifs that are important for LLPS of mouse PrP (42, 54). To study phase separation, we employed an *in vitro* system with purified proteins (42, 55, 56). This assay is based on PrP fusion proteins containing an N-terminal maltose-binding protein (MBP) to keep the recombinant proteins soluble and a C-terminal GFP to facilitate the microscopic analysis. After purification, the N-terminal MBP as well as the C-terminal His tag can be cleaved off by tobacco etch virus (TEV) protease to initiate phase transition (Fig. 1*C*). TEV-mediated release of MoN1 from MBP induced the rapid formation of highly dynamic assemblies, indicative of biomolecular condensates (Fig. 1*D* upper row) (42). XeN1 also underwent phase separation upon release of the MBP tag, but fluorescence recovery after photobleaching (FRAP) recordings revealed that the material properties of the XeN1 assemblies are different. In contrast to the liquid-like state of MoN1, the amphibian N1 was in a gel-like or aggregated state (Fig. 1*D*, lower row). These experiments suggested an essential role of the mammalian octarepeat in the ability of N1-PrP to form biomolecular condensates *via* LLPS. Interestingly, XeN1 did not remain soluble but forms irregular assemblies with a gel-like or aggregated state. Thus, the mammalian octarepeat seems to prevent a liquid-to-solid phase transition of N1-PrP by promoting LLPS and stabilizing liquid-like droplets.

### LLPS of N1-PrP is controlled by the regularly spaced tryptophan residues in the octarepeat region and not by the histidines

**Figure 2:**
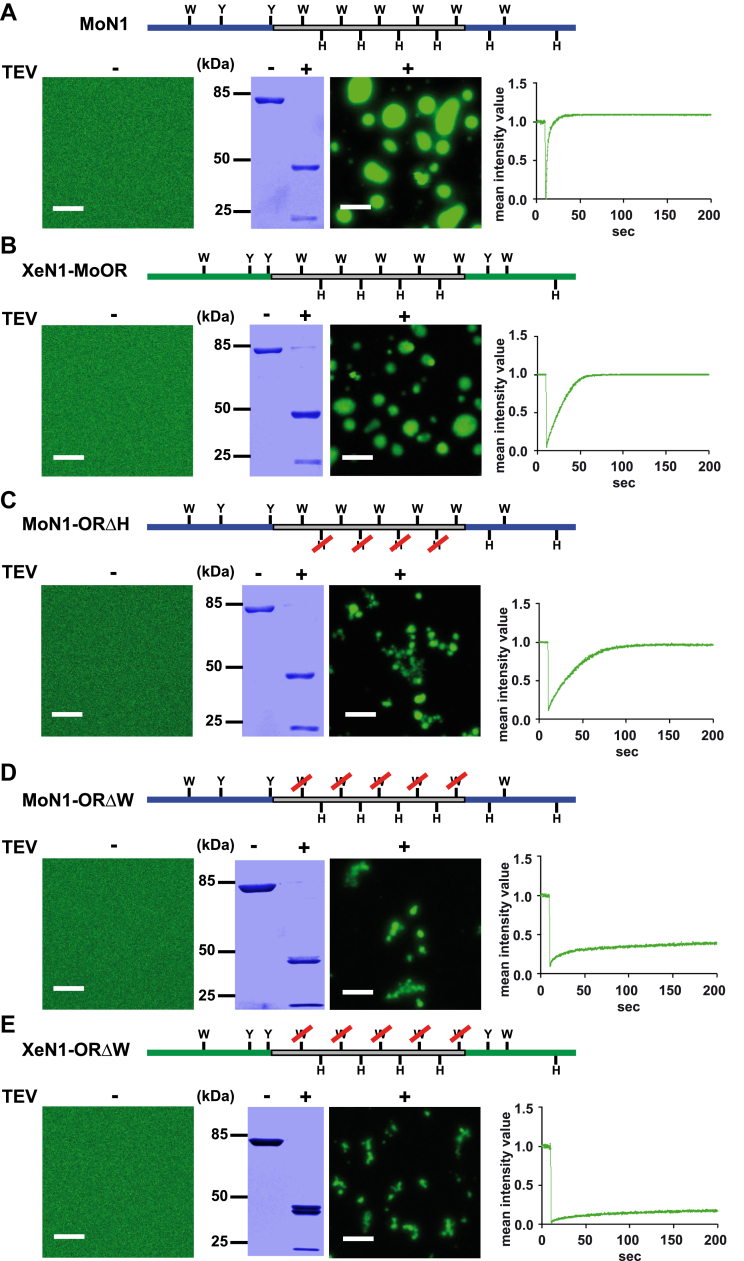
**LLPS of N1PrP is controlled by the tryptophans within the octarepeat, but not by the histidines.***A*, schematic presentation of WT MoN1 (*top* panel). Ten µM of protein in 10 mM Tris, pH 7.4 was incubated for 1 h in the absence (TEV-) or presence (TEV+) of TEV protease and then analyzed by laser scanning microscopy. The scale bar represents 10 μm. An aliquot of each sample (4.5 μg) was analyzed in parallel by SDS-PAGE and Coomassie brilliant blue staining (*middle* panel). Protein mobility within the droplets was measured by fluorescence recovery after photobleaching (FRAP) as described in Figure 1*D*. *B*, schematic presentation of XeN1-MoOR, XeN1: *green*; *graybox*: mouse octarepeat. Fluorescence microscopy and FRAP recordings were performed as described in (*A*). *C*, schematic presentation of MoN1-ORΔH. Microscopic and FRAP analyses were performed as described in (*A*). *D*, schematic presentation of MoN1-ORΔW. Fluorescence microscopy and FRAP recordings were performed as described in (*A*). *E*, schematic presentation of XeN1-ORΔW. XeN1: *green*; *gray box*: mouse octarepeat. Fluorescence microscopy and FRAP recordings were performed as described in (*A*). LLPS, liquid-liquid phase separation; TEV, tobacco etch virus.

To directly test that the octarepeat domain is causative for the differences in phase transition between mammalian and amphibian N1, we inserted the mouse octarepeat into *Xenopus* N1 (XeN1-MoOR). Indeed, the mouse octarepeat enabled amphibian N1 to form dynamic biomolecular condensates *via* LLPS. Based on FRAP recordings, it can be concluded that the XeN1-MoOR assemblies are liquid-like droplets, similarly to the the MoN1 assemblies (Fig. 2, *A* and *B*). To gain further insight into the association of protein sequence and phase behavior, we deleted conserved residues within the mammalian octarepeat. First, we replaced the four histidines in the octarepeat of mouse N1 by glycine residues. MoN1-ORΔH formed highly dynamic liquid-like assemblies, revealing that the histidines are dispensable for LLPS of MoN1 (Fig. 2*C*) (42).

Another notable feature of the mammalian octarepeat are five regularly spaced tryptophans that may promote LLPS through cation–π or π–π interactions. To test this hypothesis, we mutated and replaced five tryptophans by glycines both in mouse N1 and in the mouse-*Xenopus* N1 chimera. In contrast to MoN1 or XeN1-MoOR, MoN1-ORΔW and XeN1-ORΔW formed non-dynamic aggregates of irregular structure like amphibian N1 (Fig. 2, *D* and *E*). These findings provided the first evidence that the tryptophan residues in the mammalian octarepeat region have an important function in regulating phase transitions of PrP, whereas the histidines obviously do not play a major role in this pathway. In this context, it is interesting to note that the first repeat region that appeared in N1 during evolution is a hexarepeat in reptile PrP that contains only two histidines but already ten regularly spaced tyrosines (57). Similar to tryptophan, tyrosine can promote LLPS *via* cation–π or π–π interactions.

### LLPS of full-length PrP is critically dependent on the tryptophans in the octarepeat region, but independent of copper

**Figure 3:**
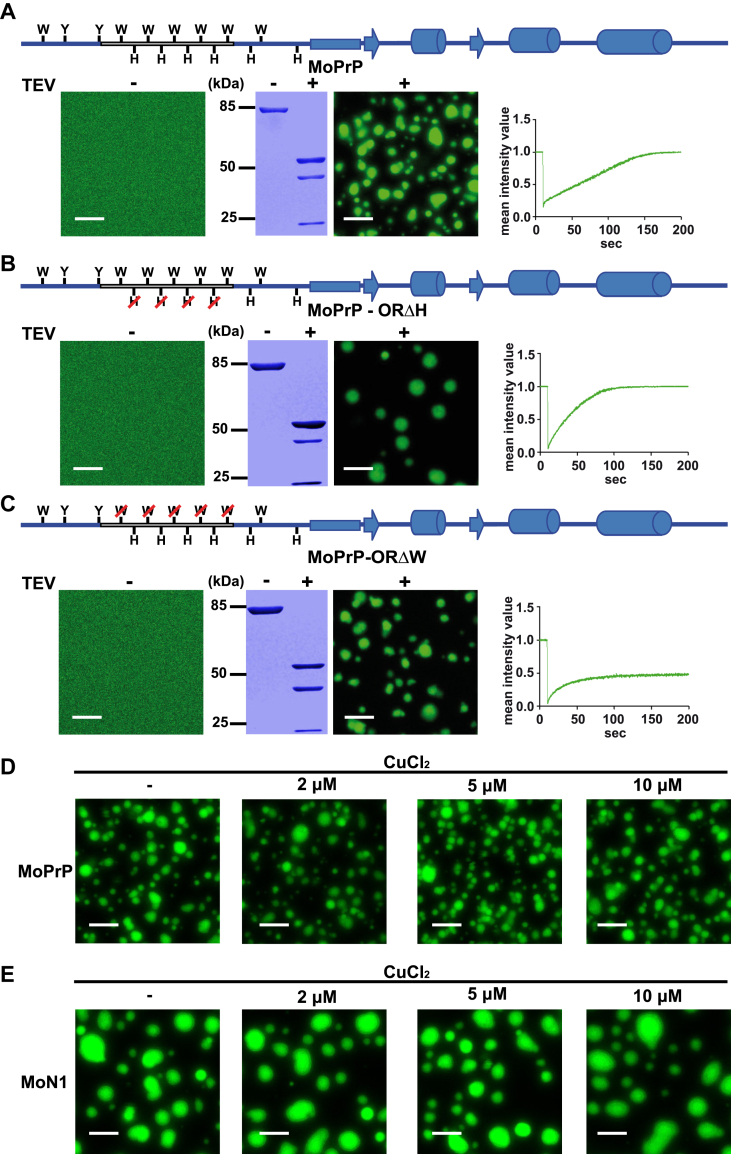
**Liquid-liquid phase separation of full-length PrP is promoted by the tryptophans in the octarepeat and not influenced by copper.***A*, schematic presentation of WT MoPrP (*top panel*). Ten µM of MoPrP in 10 mM Tris, pH 7.4 was analyzed by laser scanning microscopy before (TEV-) or after (TEV+) incubation with TEV protease for 1 h. The scale bar represents 10 μm. An aliquot of each sample (4.5 μg) was analyzed in parallel by SDS-PAGE and Coomassie brilliant blue staining (*middle panels*). Protein mobility within the droplets was measured by fluorescence recovery after photobleaching (FRAP) as described in Figure 1*D*. *B*, schematic presentation of MoPrP-ORΔH. The histidine residues in the octarepeat were replaced by glycines. Fluorescence microscopy and FRAP recordings were performed as described in (*A*). *C*, schematic presentation of MoPrP-ORΔW. The tryptophanes in the octarepeat were replaced by glycines. Fluorescence microscopy and FRAP recordings were performed as described in (*A*). *D*, ten µM of WT MoPrP in 10 mM Tris–HCl, pH 7.4 containing increasing amounts of CuCl_2_ as indicated was incubated with TEV protease for 1 h and then analyzed by laser scanning microscopy. The scale bar represents 10 μm. *E*, ten µM of WT MoN1 in 10 mM Tris–HCl, pH 7.4 containing increasing concentrations of CuCl_2_ as indicated was incubated with TEV protease for 1 h and then analyzed by laser scanning microscopy. The scale bar represents 10 μm. TEV, tobacco etch virus.

Having shown that the tryptophans, but not the histidines, within the octarepeat regulate LLPS of the N-terminally unstructured domain, we sought to verify these findings for full-length PrP. To this end, we generated two mouse PrP variants in which either the histidines (MoPrP-ORΔH) or the tryptophans (MoPrP-ORΔW) in the octarepeat were replaced by glycines. Fluorescence microscopy and FRAP recordings of phase-separated MoPrP-ORΔH and MoPrP-ORΔW confirmed the result we obtained with the isolated N-terminal domain. Replacing the histidine residues with glycines did not interfere with the LLPS of full-length MoPrP (Fig. 3, *A* *versus B*). It rather appeared that the MoPrP-ORΔH assemblies recovered faster than those formed by WT PrP. One possible explanation could be that the four missing histidine residues result in fewer π–π and cation–π interactions compared to WT PrP. This would lead to weaker intermolecular interactions and enhanced dynamicity. In contrast, the behavior of phase-separated full-length MoPrP with deleted tryptophans in the octarepeat was altered: the FRAP recordings revealed that MoPrP-ORΔW formed less dynamic gel-like or aggregated assemblies (Fig. 3*C*).

**Figure 4:**
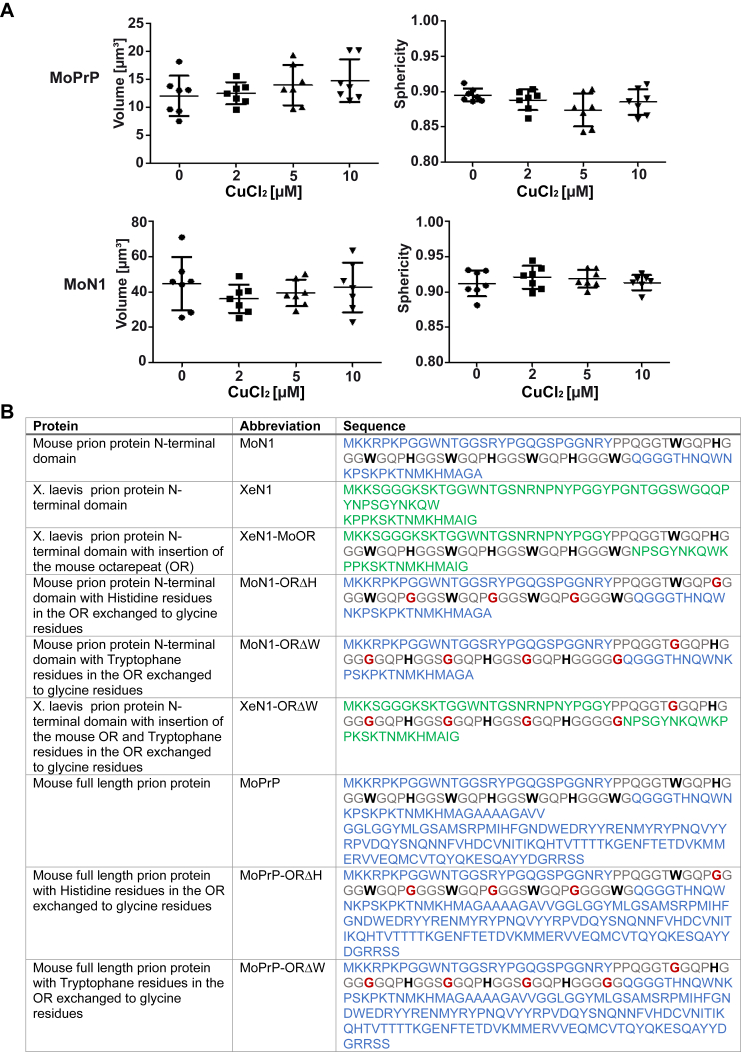
**Copper does not influence volume and sphericity of PrP condensates.***A*, image quantifications of the condensates formed by MoPrP and MoN1 shown in Figure 3, *D* and *E*. Droplet surfaces were reconstructed using Imaris 10.1.0 surface module; droplet volume and sphericity were plotted against different CuCl_2_ concentrations. Statistical analysis: Kolmogorov-Smirnov test followed by ANOVA with Dunnett’s Multiple Comparison Test (n = 7 per condition from four independent experiments). *B*, the table summarizes the names, abbreviations, and amino acid sequences of the constructs used in this study. Mouse sequences are highlighted in *blue* and *Xenopus laevis* sequences in *green*. The octarepeat is marked in *gray* and the analyzed tryptophanes and histidines are shown in *bold* letters. Mutations are depicted in *red*.

The histidines in the octarepeat domain have been shown to interact with copper. This raised the question of a possible role of copper binding in LLPS of PrP. To address this aspect, we purified WT mouse full-length PrP and N-PrP under copper-free conditions and then analyzed their phase transition in the absence or presence of 2, 5, or 10 μM CuCl_2_. Based on the microscopic analysis, the phase separation behavior of both full-length PrP and N1-PrP was not influenced by copper (Fig. 3, *D* and *E*). In addition, a quantitative analysis confirmed no differences in droplet volume and sphericity between the condensates formed with or without CuCl_2_ (Fig. 4*A*). Thus, these results suggest that copper ions are not required for LLPS of PrP and do not seem to modulate this process under physiological conditions.

Taken together, in this study, we identified a sequence-encoded molecular grammar regulating liquid–liquid phase separation of the mammalian prion proteins. First, we found that the mammalian octarepeat domain is essential for LLPS of PrP. Surprisingly, the histidines, which are specific for mammalian PrP, are dispensable for LLPS, whereas the tryptophans are critical to promote LLPS and to prevent the formation of gel-like or aggregated structures. Considering that cation–π interactions of the aromatic residues in the octarepeat with the two polybasic motifs are the main driver of LLPS, it seems plausible that tryptophans can make stronger interactions than histidines. This may be the reason why the tryptophane deletions, but not the histidine deletions, interfere with LLPS. More broadly, our study also revealed that the octarepeat domain has a biological function independent of the histidines and copper binding. This raises the intriguing question of how the activity of tryptophans to regulate phase transition is involved in the physiological activity of PrP^C^ and the formation of pathological PrP conformers.

## Experimental procedures

### Plasmids and constructs

All plasmids were maintained and amplified in *Escherichia coli* TOP10 (Thermo Fisher scientific). The utilized constructs were created by standard cloning techniques. The mouse PrP constructs are based on the coding region of mouse PrP gene (Prnp; GenBank accession number M18070) and the frog PrP constructs are based on the coding region of *X. laevis* PrP gene (7) (GenBank accession number NM_001088711.1). Both sequences were changed to express PrP-L108M/V111M (58), to enable detection by mAb3F4 (59). N1 of mouse includes aa 23 to 114; N1 of *X. laevis* includes aa 23 to 86. XeN1-MoOR: insertion of mouse octarepeat domain (OR) (aa 50–89) between *X. laevis* Y49 and N62; MoN1-ORΔH: the four H in the mouse OR were changed to G; MoN1-ORΔW: the 5 W in the OR were changed to G; XeN1-ORΔW: insertion of mouse ORΔW between *X. laevis* Y49 and N62. The names, abbreviations, and sequences of all PrP constructs analyzed in this study are also summarized in Figure 4. The MBP-TEV-PrP-eGFP-TEV-His_6_ plasmids were generated by switching the FUS coding region from pMal-TEV-FUS-eGFP-TEV-His_6_ construct, kindly provided by Dorothee Dormann (60), to the respective PrP variants.

### Protein expression and purification

Protein expression and purification was performed as previously described in (42). Shortly, MBP-N1-PrP-eGFP plasmids were transformed into BL21-DE3, and MBP-PrP-eGFP plasmids were transformed into Origami B (DE3) competent cells (Novagen). Then, 1 L of lysogeny broth medium was inoculated and grown to an absorbance (600 nm) of 0.9. Afterward, cultures were incubated for 30 min on ice. Expression was induced with 100 μM IPTG and incubated overnight at 12 °C, 120 rpm for BL21 cultures. Origami B culture expression was induced with 0.5 mM IPTG and incubated overnight at 25 °C, 120 rpm. Harvesting of bacteria was conducted at 5000*g*, 4 °C, 20 min. Subsequently, the pellet was washed with 20 ml Millipore water, centrifuged at 2000*g*, 4 °C, 20 min, and stored at −20 °C until further use. After this, the pellet was first resuspended in lysis buffer (50 mM Na_2_HPO_4_/NaH_2_PO_4_ (pH 8.0), 500 mM NaCl, 0.01 mM ZnCl_2_, 10% glycerol) and lysed with SLM AMINCO French Press (Thermo Fisher Scientific). Following, the protein solution was centrifuged at 40,000*g*, 45 min, 4 °C. A His-Trap FF column (GE Healthcare) was equilibrated with lysis buffer, then protein was loaded and washed first with five CV lysis buffer containing 20 mM imidazole and second washed with three CV lysis buffer containing 50 mM imidazole. Elution was performed by lysis buffer containing 200 mM imidazole. After elution, the protein was dialyzed overnight in dialysis buffer (50 mM Na_2_HPO_4_/NaH_2_PO_4_ (pH 8.0), 500 mM NaCl, 0.01 mM ZnCl_2_, 5% glycerol). Protein concentration was determined by NanoDrop 2000 (Thermo Fisher Scientific), aliquoted, and stored at – 80 °C. For the experiments studying the role of copper on LLPS, the purification buffers and dialysis buffer did not contain ZnCl_2_. In addition, the dialysis buffer was supplemented with 1 mM EDTA to remove any possible attached metal ions.

### Sample preparation

For sample preparation, the proteins were thawed on ice and centrifuged (20,000*g* for 10 min at 4 °C). To exchange the buffer to 10 mM Tris, pH 7.4, the solution was centrifuged (five times at 12,000*g* for 7 min at 4 °C) through Vivaspin 500 columns with 30-kDa molecular weight cut off (Sartorius Stedim biotech). Afterward, protein concentration was determined by NanoDrop 2000. Phase transition was initiated by adding TEV protease for 1 h to the samples. To analyze the effect of copper on PrP phase separation, we used a buffer with high-purity Tris-HCl from PanReac AppliChem. In addition, before every experiment, the protein solutions were tested for copper contamination with the Copper Assay Kit from Sigma-Aldrich. When no copper was detected with this kit, the corresponding protein solution was defined as copper free. To study the effect of increasing copper concentrations on LLPS, CuCl_2_ was added to the reaction before TEV protease.

### Laser scanning microscopy

Fluorescent imaging laser scanning microscopy was performed as described in (42). Imaging was conducted on an ELYRA PS.1 (Carl Zeiss) microscope with an imaging detector (LSM 880; Carl Zeiss). The 63x numerical aperture 1.4 oil-immersion objective was utilized to record a z-stack of 67.5 × 67.5 × 10 μm and 0.900 μm for each optical section. The power of the argon laser was set to 0.006% at 488 nm with pixel dwell time of 5.71 μs. All settings were kept constant during the measurements. FRAP experiments were performed with the Plan-Apochromat 100x numerical aperture 1.46 oil differential interference contrast M27 objective and ZEN2.1 bleaching and region software module. Three circular regions with a 12-pixel diameter were utilized as regions of interest. Two regions were deployed as background signal and reference signal. The other region was bleached with 100% laser power and a pixel dwell time of 8.71 ms, with scan time of 111.29 ms and pixel dwell time of 1.61 ms. Excel 2016 was utilized for data evaluation and diagrams were designed in GraphPad Prism.

## Data availability

All data are contained within the manuscript.

## Conflict of interest

The authors declare that they have no conflicts of interest with the contents of this article.
